# Supplementary material for: Polychoric Correlation With Ordinal Data in Nursing Research
Source: Nurs Res. 2022 Aug 20;71(6):469–76. doi: 10.1097/NNR.0000000000000614 (PMC9617753; doi:10.1097/NNR.0000000000000614)
Supplement: SUPPLEMENTARY MATERIAL [file nres-71-469-s001.docx]

Running head: Polychoric Correlation

**Polychoric Correlation with Ordinal Data in Nursing Research**

Frank Kiwanuka^1^, Juho Kopra^1^, Natalia Sak-Dankosky^2^, Rose Clarke Nanyonga^3^, Tarja Kvist^1^

^1^University of Eastern Finland, Department of Nursing Science, Faculty of Health Sciences, Kuopio, Finland

^2^Medical University of Warsaw, Department of Clinical Nursing, Warsaw, Poland

^3^Clarke International University, Kampala, Uganda

**Author Note**

Frank Kiwanuka
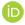
https://orcid.org/0000-0001-8178-3120

Juho Kopra
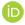
https://orcid.org/0000-0002-2140-8027

Natalia Sak-Dankosky
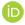
https://orcid.org/0000-0002-2140-8027

Rose Clarke Nanyonga
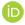
https://orcid.org/0000-0001-7709-5667

Tarja Kvist
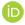
https://orcid.org/0000-0001-5974-8732

Research reported in this publication was supported by grants from the Finnish National Agency for Education, the North Savo Cultural Fund and the Kuopio University Foundation. Rose Clarke Nanyonga was supported in part with grants from the Fogarty International Center of the National Institutes of Health and the Office of the U.S. Global AIDS Coordinator and Health Diplomacy, the U. S. President’s Emergency Plan for AIDS Relief (Grant No. 1R25TW011213). The content is solely the authors’ responsibility and does not necessarily represent the official views of the funders.

The University of Eastern Finland, Committee on Research Ethics and the Research and Ethics Committee of Clarke International University Ethical provide ethical approval. The study was registered with the Uganda National Council of Science and Technology. Approval to collect data from the setting was received from the administration of the study settings.

The authors have no conflicts of interest to report.

Corresponding author: Frank Kiwanuka MSc, CNS, RN, Department of Nursing Science, Faculty of Health Sciences, University of Eastern Finland, 70211, Kuopio, Finland. Email: franki@uef.fi

# Abstract

***Background*:** Measures in nursing research frequently use Likert scales that yield ordinal data. Confirmatory factor analysis using Pearson correlations commonly applies to such data, although this violates ordinal scale assumptions.

***Objectives*:** To illustrate the application of polychoric correlations and polychoric confirmatory factor analysis as a valid alternative statistical approach using data on family members’ perceived support from nurses as an exemplar.

***Methods*:** A primary analysis of cross-sectional data from a sample of 800 participants using data collected with the Icelandic-Family Perceived Support Questionnaire was conducted using polychoric versus Pearson correlations, analysis of variance, and confirmatory factor analysis.

***Results*:** A two-factor measurement model was compatible with data from family members in the Ugandan care settings. Two contextual factors (cognitive and emotional support) constituted the family support measurement model. A factor correlation indicated that the two factors reflected distinct but closely related aspects of family support. Polychoric correlation revealed 13.8% (range 5.5%–25.2%) higher correlations compared to Pearson correlations. Moreover, the polychoric agreed with the data, while the Pearson confirmatory factor analysis did not fit based on multiple statistical criteria. Analyses indicated a difference in emotional and cognitive support perception across two family characteristics: education and relationship to the patient.

***Discussion*:** A polychoric correlation suggests stronger associations, and consequently, the approach can be more credible with an ordinal Likert scale than Pearson correlations. Hence, polychoric confirmatory factor analysis can address a larger proportion of variance. In nursing research, polychoric confirmatory factor analysis can confidently be utilized when conducting confirmatory factor analysis of ordinal variables in Likert scales. Further, when a Pearson confirmatory factor analysis is used for ordinal Likert scales, the researcher should carefully evaluate the difference between the two approaches and justify their methodological choice. Even though we do not suggest dispensing with Pearson correlations entirely, we recommend using polychoric correlation for ordinal Likert scales.

*Keywords:* confirmatory factor analysis, family nursing, Pearson correlations, polychoric correlation

**Polychoric Correlation with Ordinal Data in Nursing Research**

# Likert scales (e.g., ordinal scales) are very common across nursing research; such scales are multi-item instruments with graded-response items (Lorenzo-Seva & Ferrando, 2015). Ordinal scales are categorical, with ordered possible values (e.g., a 5-option scale varying from “almost always” to “almost never”) for which the distance between subsequent options is not necessarily similar. In the past, some researchers have argued that more accurate measurement models can be obtained by using polychoric correlations for ordinal data generated from Likert scales (see Holgado-Tello et al., 2010). Other scholars have argued that variables within the ordinal data can be assumed as continuous where the variables are considered a set number of ordered categories (Hu & Bentler, 1999); thus, the frequent application of Pearson correlations to confirmatory factor analysis (CFA). However, Pearson correlations assume unrealistically a multivariate normal distribution (MVN), while the polychoric correlation assumes a latent MVN for which the borders of ordinal classes are estimated. Of note is that statistical interpretations are based on diverse methods—with each method having its own assumptions.

# In nursing research on family support from nurses, various studies (e.g., Bruce et al., 2016; Dieperink et al., 2018; Konradsen et al., 2018) have commonly used the Icelandic-Family Perceived Support Questionnaire (ICE-FPSQ: Sveinbjarnardottir et al., 2012). The ICE-FPSQ was developed using the conceptual framework set out in the Calgary Family Intervention Model (CFIM). According to the CFIM, family support is directed at three domains of family functioning: cognitive, emotional, and behavioral (Wright & Leahey, 2009). Psychometric testing of the ICE-FPSQ instrument showed that it assesses two domains: emotional and cognitive support (Sveinbjarnardottir et al., 2012). Traditionally, variables on the ICE-FPSQ tool are scored on a Likert scale, thus providing ordinal data, although, given the nature of the ICE-FPSQ scale, some nursing researchers (e.g., Bruce et al., 2016; Konradsen et al., 2018; Sveinbjarnardottir et al., 2012) have conducted Pearson correlations for their factor analysis. It is worth assessing the predictive accuracy and application of alternative statistical approaches in nursing research. A large sample (*n* = 800) was used to evaluate the differences between Pearson CFA and polychoric CFA in this study. Most statistical software can fit CFA models only based on Pearson correlations. In contrast, polychoric CFA can be conducted in Mplus (Muthen & Muthen, 2017), R programming (R-Core Team, 2020) in a lavaan package (Rosseel, 2012), and the “FACTOR” software (Ferrando & Lorenzo-Seva, 2017).

# Polychoric correlation considers and sorts variables into a series of categories; it is an alternative to the Pearson coefficient, specifically for cases where instruments yield ordinal data from categorical variables (Jin & Cao, 2018). The Pearson CFA uses the maximum likelihood (ML), which assumes that observed data follow a continuous-multivariate normal distribution. This assumption does not fit ordinal observed variables (Li, 2016). According to Byrne (2012, p. 129), the correlations are underestimated when categorical data are treated as continuous (as in the Pearson CFA). The continuous approach is violated, particularly when data has high skewness and especially when the two variables are skewed in opposite directions (Bollen & Barb, 1981). Skewness also inflates the chi-squared value leading to an unrealistically small *p*-value on chi-squared tests of fit. Thus, it seems clear that alternative statistical criteria should be considered for model fit evaluation. However, nursing scientists should be cautious when treating Likert data as continuous data. If the data are skewed, and the number of categories is small (< 5), then CFA based on a Pearson correlation affects the estimates—increasing the number of categories and utilizing other approaches, such as polychoric CFA, can be considered in these circumstances (Byrne, 2012).

# Polychoric CFA is fitted to data with weight least squares, means, and variances (WLSMV), specifically designed for ordinal data, and produces acceptable model–data fit indices for evaluating the proposed model (DiStefano & Morgan, 2014). Various scholars (e.g., Finney & DiStefano, 2006; Holgado-Tello et al., 2010) have argued that polychoric correlations are more accurate and offer more robust estimations when handling ordinal data originating from Likert scales. More recently, Jin and Cao (2018) also recommended using polychoric correlations for Likert data. However, despite these researchers’ strong arguments, few applied resources currently use this approach in nursing research. In these circumstances, we believe that paying close attention to conducive and alternative analytical methods has great potential to advance nursing research and explain seemingly inconsistent results.

**Background of Ugandan Study**

# The current study examined hospital patients’ families and their perceived support from nurses. Although nurses are well-positioned to support family members using various interventions (Kiwanuka et al., 2022), it is noticeable that support during hospitalization of a patient is disproportionately perceived across studies. Dieperink et al. (2018) reported that Danish and Australian families reported low emotional support from nurses in Denmark and Australia. Similarly, we find that in Sweden, the parents of children with congenital heart defects indicated that nurses in pediatric outpatient clinics offered low levels of family support (Bruce et al., 2016). Meanwhile, African researchers are beginning to understand the importance of families in care: Imanipour and Kiwanuka (2020) reported that although nurses consider the family a crucial entity of effective care, their support is minimal. The family perspective is undoubtedly needed if we are to develop effective strategies for supporting the family while utilizing robust analytical approaches.

# Two main questions guided analyses to illustrate the application of polychoric correlation and polychoric CFA as alternative statistical approaches to the Pearson CFA, using data on family members’ perceived support from nurses as an exemplar:

# What are the benefits of using polychoric CFA instead of the more commonly applied Pearson CFA?

- What associations exist between background characteristics and family members’ perceived emotional and cognitive support?

# Methods

The study targeted the family members of hospitalized patients; more specifically, family members who were at least 18 years old were eligible to participate. Here, “family members” were defined as whoever the patient identified as their “family,” a definition that certainly included blood relatives but was not limited to those with a common ancestral background; close friends, for instance, were included. The study data were collected using a self-administered paper questionnaire distributed at two Uganda hospitals from June 2020 to August 2020. The study utilized a cross-sectional design using convenience sampling. The two study settings were the national referral hospital of Uganda (> 1,000-bed capacity with about 1,500 nursing staff) and the largest private for-profit hospital (100-bed capacity with about 150 nursing staff). The data were collected from adult medical, surgical, obstetrics, and gynecology wards. Outpatient units were excluded because the target population comprised family members of adult hospitalized patients. The study was approved by a Finnish University Committee on Research Ethics (*Statement 3/2020*) and a Ugandan hospital’s research and ethics committee. Lastly, clearance was obtained from the administrations of the two study settings before commencing data collection.

**Measures**

Background characteristics, gathered using a sociodemographic form, included the following: gender, a dichotomic variable (female or male); setting, a categorical variable (Hospital I and Hospital II); age, a variable with continuous observations; education, a categorical variable (none, primary, secondary, and postsecondary); relationship with the patient, a categorical variable (spouse [wife/husband], child, parent, grandparent, surrogate, other); and marital status, a categorical variable with several options (married, single, cohabiting, or widowed).

The ICE-FPSQ was used to collect data about family members’ perceived support from nurses when a family member is experiencing illness. The tool was originally developed in Iceland using a sample of family members based on the CFIM mentioned above (Sveinbjarnardottir et al., 2012). The tool measures a family’s perceptions of support provided by nurses and the effect of family nursing interventions. The ICE-FPSQ contains 14 items in two subsections assessing: (a) cognitive support (five items, α = .881); and (b) emotional support (nine items, α = .952). The tool has good psychometric properties, with a total Cronbach’s alpha of .961. The scoring of the tool is based on a 5-option ordinal Likert scale varying from “almost always” (5) to “almost never” (1). The total score ranges from 14 (minimum) to 50 (maximum), with higher scores reflecting more family support (Sveinbjarnardottir et al., 2012).

### *Translation*

The ICE-FPSQ was distributed either in English (the source language) or the Luganda language (the target language). While recruiting family members, literacy in either English or Luganda was considered. Cross-cultural adoption and the protocol for translation were conducted using the “back-translation” (Brislin, 1970) approach. The back-translation is a widely recommended method when the meaning of the contents of an instrument (e.g., phrases used in the ICE-FPSQ) do not necessarily imply precisely the same thing in another language. Back-translation allowed for a functionally equivalent translation where the original and target versions are evaluated (Brislin, 1970).

A bilingual nurse with a good understanding of the content of the ICE-FPSQ and the target population (i.e., family members) translated the ICE-FPSQ into Luganda. One family member, a layperson, and a native user of the target language assessed what each item of the translated version meant. The first author was involved at all stages to ensure that the instrument structure and the meaning of items were maintained. Three nurses then back-translated the Luganda version into the source language and compared the two versions in the source language while highlighting discrepancies.

The content validity indices (CVIs; Polit & Beck, 2006) of the ICE-FPSQ were computed for the current study. Five reviewers who were experienced nurses in family health care, conducted relevance rating of item content using a 4-point scale (1 = highly relevant; 2 = quite relevant; 3 = somewhat relevant; 4 = not relevant). Note that a 4-point scale is recommended since it avoids an ambivalent midpoint score (Lynn, 1986). The proportional item relevance rating for the five reviewers across all items on the ICE-FPSQ was assessed using the universal agreement (UA) method. The UA among experts was 1; this indicated an acceptable CVI for three to five reviewers.

## Statistical Analysis

Before collecting the data, the sample size was calculated using power analysis based on data from the study of Sveinbjarnardottir et al. (2012): A statistical power of 80% and a 5% level of confidence were considered, indicating a minimum sample size of 394 participants. Survey responses were analyzed using R Statistical Software (v4.1.2; R Core Team 2021) with R packages psych (Revelle, 2022) and lavaan (Rosseel, 2012).

Descriptive statistics including frequency, mean, and standard deviations were computed for demographic characteristics of the participants. Pearson and polychoric correlation matrices and skewness were calculated for 14 variables for inclusion in the CFA.

Both the Pearson CFA and polychoric CFA were fitted to the data. The CFA model was based on the Icelandic study of Sveinbjarnardottir et al. (2012). The first five items were set to the cognitive support factor, and the last nine items formed the emotional support factor. To ensure the model identifiability, the first loadings for both factors were set to 1.

Model fit was evaluated using multiple statistical criteria with conventional cut-off values, so that root mean square error approximation (RMSEA) < 0.06 for *n* ≥ 250, comparative fit index (CFI) > 0.95, Tucker Lewis index (TLI) > 0.95, and standardized root mean square residual (SRMR) < 0.11 supported the model fit (Hu & Bentler, 1999). Tucker’s congruence coefficient (Lorenzo-Seva & Ten Berge, 2006) was calculated to evaluate the similarity between the factor solutions of the two methods. Correlation residuals were calculated to indicate which item correlations fit well with CFA models. Since the instrument was being used for the first time in the setting, Cronbach’s alpha values were computed to assess the internal consistency of the ICE-FPSQ.

Factor scores were calculated based on the CFA method, which was better supported by the aforementioned statistical criteria. Item-specific Cronbach’s alphas were calculated for both CFA methods. Also, analysis of variance (ANOVA) and visual inspection was conducted for factor scores to explore differences in perception of emotional and cognitive support across family characteristics.

**RESULTS**

**Data Overview**

Of 850 questionnaires distributed, 810 recipients responded. Ten (1.2%) of the 810 questionnaires were omitted from analysis because more than five items were missing from the entire questionnaire. Thus, 800 responses were deemed suitable and included in the analysis. A majority of the family members identified as the patient’s siblings (*n* = 366)*.* Other categories included parents (father or mother, *n* = 124), friend (*n* = 113), patient’s child (*n* = 73), spouse (husband or wife *n* = 38), grandparent (*n* = 21), surrogates (*n* = 20), and other unspecified relatives (*n* = 45). Three quarters of the participants (*n* = 600) were from a tax-funded public health care setting, while a quarter (*n* = 200) were from a private for-profit healthcare setting. The age of participants ranged from 18 to 72 years, with a mean age of 26 years (*SD* = 7.45 years). Approximately 63% (*n* = 504) were female while 37% (*n* = 295) were male. Most (73.7%) family members had completed postsecondary education (e.g., vocational training or university). Average skewness of the 14-item questions was -0.315 (minimum–maximum -0.586 to -0.016). Table 1 shows polychoric (values below the matrix diagonal) and Pearson correlations (values above the matrix diagonal). Analyses indicated a significant difference between the two approaches. Of note is that the use of polychoric correlations gives, on average, 13.8% higher correlations with our data (range lower bound 5.5%, upper bound 25.2%).

# Applying Polychoric Correlation to Confirmatory Factor Analysis

We completed two overarching analyses as part of the illustration: (a) comparison of polychoric and Pearson CFAs, and (b) evaluation of variance of family support. The analysis considered 14 different items on the ICE-FPSQ. The previous study informed the family support measurement model of Sveinbjarnardottir et al. (2012). In keeping with prior knowledge (e.g., Bruce et al., 2016; Konradsen et al., 2018; Sveinbjarnadottir et al., 2012), the family support measurement model included two contextual factors (cognitive support and emotional support).

All factor loadings were higher than the cut-off of 0.6 (Field, 2005), thus supporting convergent validity (Supplemental Digital Content [SDC] Table 1, http://links.lww.com/NRES/A443). The ICE-FPSQ in this study had a total Cronbach’s alpha of .89, .87 for the emotional support subscale, and .74 for the cognitive support subscale, respectively. Item-specific Cronbach’s alpha values for the Pearson’s CFA ranged from .88 to .891, while those of the polychoric CFA ranged from .901–.91. The item-specific difference between ordinal Pearson and polychoric ordinal alphas ranged from .019 to .020 (SDC Table 2).

Chi-square tests showed significant *p*-values, which would indicate that the model does not fit. Chi-squared *p*-values were expected to be unrealistically small due to the large amount of data (and skewness). Thus, other fit indices such as RMSEA, SRMR, TLI, and CFI were chosen to evaluate model fit. RMSEA values were not satisfactory for either of the CFA approaches. Regarding Pearson CFA, the only satisfactory fit indicator was SRMR, while for polychoric CFA, the SRMR, TLI, and CFI indices supported the model at least a satisfactory level (Table 2).

A factor congruence coefficient of .91 for cognitive support between Pearson and polychoric CFA indicates fair similarity (.85–.94), but they should not be considered equal (> .95; Lorenzo-Seva &Ten Berge, 2006). Regarding congruence of emotional support, the value .96 indicates that the solutions provided by the two methods are equal. There was no evidence of similarity between different factors (congruence < .01 and .12).

**Associations Between Family Members’ Background Characteristics and Their Perceived Emotional and Cognitive Support**

The results of testing potential variance of family support across characteristics of the sample are presented in Table 3. ANOVA indicated a difference in perception of emotional and cognitive support across two family characteristics: education and relationship to the patient. To expand on the understanding of relationships among variables in the model, we further conducted analyses of significant variables to identify any interactions (SDC Table 3). Education consisted of four levels (*none, primary, secondary, postsecondary, and other*). At the same time, the relationship with the patient constituted 11 responses (*mother, father, sibling, wife, husband, friend, surrogate carer, hired carer, grandparent, child of patient, and other relatives*).

Consequently, analyses indicated that the perceived cognitive or emotional support is different in different education categories and among the different relationship groups of the family member to the patient. For example, family members with secondary/college education—among whom are the categories parents, spouses, friends, surrogate carers, and children—the experience is one of *positive* cognitive and emotional support (interactions *p* < .05). In contrast, siblings and grandparents seem to experience *negative* cognitive and emotional support (interactions *p* < .05). Further, family members with postsecondary education (from, e.g., tertiary institutions such as universities) who are related to the patient in any of these categories (i.e., father, wife, hired carer, or grandparent) seem to experience positive cognitive and emotional support (interactions *p* < .05). Likewise, for family members with no formal education, only siblings share positive cognitive and emotional support (*p* < .05). Family members with primary education and related to the patient as surrogates, hired carers, grandparents, or children experience positive cognitive or emotional support relative to other categories (*p* < .05).

**Discussion**

This analysis shows a favorable estimation of CFA with polychoric correlation in the context of observed ordinal variables versus CFA with Pearson correlations. The analyses focused mainly on the application and benefits of polychoric correlations applied to the use of CFA using 5-item Likert scale data. Outcomes of comparative analyses indicate that polychoric correlations’ results differ from those obtained using Pearson correlations. The study analyses indicate that the observed patterns were close. Still, the polychoric approach was supported by four out of five model fit indices, while the Pearson counterpart was supported by only one. As a result, the two approaches suggest similar factor solutions for this data set.

Nonetheless, the polychoric correlation obtains higher correlation measures, and therefore a larger proportion of variance can be explained after factor analysis. This finding is in line with Holgado-Tello et al. (2010), implying that a more accurate measurement model can be generated when polychoric correlations are used for Likert scale data. Although it is not known if either of the two CFA approaches generated the data, the model fit indices show that polychoric CFA yields results that describe the data better. Polychoric CFA also requires less restrictive assumptions than would be the case when assuming a continuous scale for Likert scale data. It means that the usage of polychoric CFA can be seen as a safer way to analyze Likert scale data because of its robustness against assumptions that could restrict the use of another method. Some scholars have argued that if there are five or more item response options (e.g., as in the ICE-FPSQ), traditional Pearson correlation ML-based methods with adjustments can be used. However, relying solely on traditional ML methods minimizes the categorical nature of data and exposes the misspecification of models.

Our data were obtained with the ICE-FPSQ questionnaire that has been used in other studies (e.g., Bruce et al., 2016; Konradsen et al., 2018; Sveinbjarnadottir et al., 2012). Although the questionnaire is the same, it is noteworthy that the polychoric correlation approach is a significant departure from previous studies that have used the Pearson correlation with data from the ICE-FPSQ and other studies using Likert scales in nursing research. The Pearson correlation requires that variables have at least an interval scale, while Likert variables are ordinal. Although Pearson correlations for Likert variables with ordinal data are common, it seems to be a frequent methodological misstep that requires justification when applied in practice. Finney and DiStefano (2006) gave easily understandable recommendations for using these statistical methods, while Byrne (2012) described issues caused by their misusage. These arguments for and against two important statistical methods stimulated our interest in researching which method would work better in our particular research context.

We recommend polychoric correlations available in R package lavaan against Pearson correlations when conducting a CFA of ordinal variables using Likert scales in nursing research. While nursing researchers commonly use Likert scales yielding ordinal data, most are well-versed in using Pearson correlations to perform factor analysis using SPSS. Polychoric correlation can, as mentioned, be conducted with R package lavaan but is not available in SPSS, a factor that makes the choice of analytics software critical. Recognizing that our data, as well as that presented in other nursing science studies (e.g., Bruce et al., 2016; Konradsen et al., 2018; Sveinbjarnardottir et al., 2012), is from a questionnaire with an ordinal scale, the most suitable approach for analyzing the data must be chosen.

Dimensionality evaluation of a scale in other settings is essential for reviewing the psychometric properties of existing ordinal scales. In this context, and using a large sample size (*n* = 800) and a Likert scale (i.e., the ICE-FPSQ), we illustrate significant differences in results when using the two approaches. In particular, analyses from our data indicate that polychoric CFA fitted the data better than the Pearson correlation CFA model. To shed light on potential differences in the threshold values indicating model fit (e.g., RMSEA, SRMR, CFI, TLI), we adopted the cut-off values suggested by Hu and Bentler (1999). A chi-square test is often significant in CFA models for real-world data of this size (*n* = 800). Thus, using a chi-squared *p*-value is not an excellent way to assess how well the model fits the data. Other indices are more reliable in determining the model fit for large data.

The two approaches considered here seem to play an important role concerning the fit indices and misspecification. This further suggests the importance of carefully evaluating the difference between the two approaches to justify the methodological choice for ordinal Likert scales. In the current study, we used guidance from several important prior publications to demonstrate that the ICE-FPSQ can be used in the setting. This strengthens the ability of the measures (i.e., items on the ICE-FPSQ) to capture change related to interventions designed for supporting families in the setting. Accordingly, the location and scale variables were kept at the default factors proposed by the initial model of Sveinbjarnadottir et al. (2012).

Participants’ scores of perceptions of the cognitive and emotional support received from nurses suggest that nurses in the study settings did offer family support at sufficient levels. This finding can plausibly be attributed to the significance of the family as an essential element of the care provided by nurses—something which was revealed in the same setting in an earlier study (Imanipour & Kiwanuka, 2020). Elsewhere in the literature, this finding contradicts results reported from a study among Swedish families who endorsed lower response options on the ICE-FPSQ (Bruce et al., 2016).

**Strengths and Limitations**

The use of a relatively large sample size, 800 family members, denotes a particular strength of the study, as it provides extensive data that is likely to answer the research questions. The use of polychoric correlation is well suited to ordinal data drawn from a Likert scale; thus, we are in a good position to examine the assertion raised by other scholars (e.g., Jin & Cao, 2018) that polychoric correlations are more accurate and offer more robust estimations when handling ordinal data originating from Likert scales. Another strength of the study is the inclusion of family members from units where the illness experience at the time of admission of a patient provides substantial family perceived support measures from nurses. Overall, this article contributes to the compelling arguments for using the polychoric correlations approach to CFA with ordinal data compared to Pearson correlations. Certainly, Pearson correlation is the primary way researchers have yet conducted CFA with ordinal data from Likert scales. However, the current study’s robust methodological work should convince researchers in nursing science that choosing polychoric correlation CFA for ordinal data can be justified.

There is one notable limitation to this study. When using a scale that is not well validated in specific settings, it can be necessary to predict the frequency of individual item-level responses and items scrutinized before data collection. This was not done in this study, however, and hence predicting the frequency of individual item responses is challenging.

**Conclusion**

Our data show that choosing a polychoric correlations approach to CFA with ordinal data compared to Pearson correlations seems to significantly affect the compatibility of a two-factor model. We recommend that nurse researchers can utilize the polychoric correlation for factor analysis. This approach is statistically more robust than the traditional approach—albeit often overlooked. Given the frequent use of Likert scales that yield ordinal data, it would be interesting for nurse researchers to investigate polychoric CFA models of ordinal variables using existing software, such as lavaan or Mplus.

# References

Bollen, K. A., & Barb, K. H. (1981). Pearson’s r and coarsely categorized measures. *American Sociological Review*, *46*, 232-239. https://doi.org/10.2307/2094981

Brislin, R. W. (1970). Back-translation for cross-cultural research*. Journal of Cross-Cultural Psychology*, *1*, 185-216. https://doi.org/10.1177/135910457000100301

Bruce, E., Dorell, Å., Lindh, V., Erlingsson, C., Lindkvist, M., & Sundin, K. (2016). Translation and testing of the Swedish version of Iceland-family perceived support questionnaire with parents of children with congenital heart defects. *Journal of Family Nursing*, *22*, 298-320. https://doi.org/10.1177/1074840716656343

Byrne, B. M. (2012). *Structural equation modeling with Mplus: Basic concepts, applications, and programming*. Routledge.

Dieperink, K. B., Coyne, E., Creedy, D. K., & Østergaard, B. (2018). Family functioning and perceived support from nurses during cancer treatment among Danish and Australian patients and their families. *Journal of Clinical Nursing*, *27*, e154-e161. https://doi.org/10.1111/jocn.13894

DiStefano, C., & Morgan, G. B. (2014). A comparison of diagonal weighted least squares robust estimation techniques for ordinal data. *Structural Equation Modeling: A Multidisciplinary Journal*, *21*, 425-438. https://doi.org/10.1080/10705511.2014.915373

Ferrando, P. J., & Lorenzo-Seva, U. (2017). Program FACTOR at 10: Origins, development and future directions. *Psicothema*, *29*, 236-240. https://doi.org/10.7334/psicothema2016.304

Field, A. (2005). *Discovering statistics using SPSS* (2nd ed.). SAGE Publications.

Finney, S. J., & DiStefano, C. (2006). Non-normal and categorical data in structural equation modeling. In G. R. Hancock & R. O. Mueller (Eds.), *Structural equation modeling: A second course* (pp. 269-314). Information Age Publishing Inc.

Holgado-Tello, F. P., Chacón-Moscoso, S., Barbero-García, I., Vila-Abad, E. (2010). Polychoric versus Pearson correlations in exploratory and confirmatory factor analysis of ordinal variables. *Quality & Quantity*, *44*, 153. https://doi.org/10.1007/s11135-008-9190-y

Hu, L.-T., & Bentler, P. M. (1999). Cutoff criteria for fit indexes in covariance structure analysis: Conventional criteria versus new alternatives. *Structural Equation Modeling: A Multidisciplinary Journal*, *6*, 1-55. https://doi.org/10.1080/10705519909540118

Imanipour, M., & Kiwanuka, F. (2020). Family nursing practice and family importance in care–Attitudes of nurses working in intensive care units. *International Journal of Africa Nursing Sciences*, *13*, 100265. https://doi.org/10.1016/j.ijans.2020.100265

Jin, S., & Cao, C. (2018). Selecting polychoric instrumental variables in confirmatory factor analysis: An alternative specification test and effects of instrumental variables. *British Journal of Mathematical and Statistical Psychology*, *71*, 387-413. https://doi.org/10.1111/bmsp.12128

Kiwanuka, F., Sak-Dankosky, N., Alemayehu, Y. H., Nanyonga, R. C., & Kvist, T. (2022). The evidence base of nurse-led family interventions for improving family outcomes in adult critical care settings: A mixed method systematic review: Nurse-led family interventions in Intensive Care. *International Journal of Nursing Studies*, *125*, 104100. https://doi.org/10.1016/j.ijnurstu.2021.104100

Konradsen, H., Dieperink, K. B., Lauridsen, J., Sorknaes, A. D., & Ostergaard, B. (2018). Validity and reliability of the Danish version of the Ice Expressive Family Functioning and Ice Family Perceived Support questionnaires. *Scandinavian Journal of Caring Sciences*, *32*, 1447-1457. https://doi.org/10.1111/scs.12591

Li, C. H. (2016). Confirmatory factor analysis with ordinal data: Comparing robust maximum likelihood and diagonally weighted least squares. *Behavior Research Methods*, *48*, 936-949. https://doi.org/10.3758/s13428-015-0619-7

Lorenzo-Seva, U., & Ferrando, P. J. (2015). POLYMAT-C: A comprehensive SPSS program for computing the polychoric correlation matrix. *Behavior Research Methods*, *47*, 884-889. https://doi.org/10.3758/s13428-014-0511-x

Lorenzo-Seva, U., & Ten Berge, J. M. F. (2006). Tucker’s congruence coefficient as a meaningful index of factor similarity. *Methodology*, *2*, 57-64. https://doi.org/10.1027/1614-2241.2.2.57

Lynn, M. R. (1986). Determination and quantification of content validity. *Nursing Research*, *35*, 382-386. https://doi.org/10.1097/00006199-198611000-00017

Muthén, B., & Muthén, L. K. (2017). *Mplus version 8 user’s guide* (pp. 507-518). Muthén & Muthén.

Polit, D. F., & Beck, C. T. (2006). The content validity index: Are you sure you know what’s being reported? Critique and recommendations. *Research in Nursing & Health*, *29*, 489-497. https://doi.org/10.1002/nur.20147

R Core Team. (2021). R: A language and environment for statistical computing. R Foundation for Statistical Computing. Retrieved January 3, 2021, from https://www.r-project.org

Revelle, W. (2022). psych: Procedures for psychological, psychometric, and personality research. Northwestern University, Evanston, Ill. R package version 2.2.5, <https://CRAN.R-project.org/package=psych>

Rosseel, Y. (2012). lavaan: An R package for structural equation modeling. Journal of Statistical Software, *48*, 1-36. http://www.jstatsoft.org/v48/i02/

Sveinbjarnardottir, E. K., Svavarsdottir, E. K., & Hrafnkelsson, B. (2012). Psychometric development of the Iceland-family perceived support questionnaire (ICE-FPSQ). *Journal of Family Nursing*, *18*, 328-352. https://doi.org/10.1177/1074840712449203

Wright, L. M., & Leahey, M. (2009). *Nurses and families: A guide to family assessment and intervention* (5th ed.). F. A. Davis.

Table 1

*Matrix of Polychoric Correlations (below) and Pearson Correlations (above) for the ICE-FPSQ Scale*

| Items |  | | | | | | | | | | | | | |
| --- | --- | --- | --- | --- | --- | --- | --- | --- | --- | --- | --- | --- | --- | --- |
|  | I | II | III | IV | V | VI | VII | VIII | IX | X | XI | XII | XIII | XIV |
| I | 1.00 | .43 | .32 | .36 | .22 | .34 | .45 | .46 | .24 | .29 | .44 | .32 | 45 | .38 |
| II | .47 | 1.00 | .49 | .30 | .39 | .34 | .38 | .45 | .43 | .47 | .28 | .25 | .27 | .26 |
| III | .36 | .53 | 1.00 | .38 | .30 | .39 | .41 | .36 | .33 | .40 | .37 | .12 | .36 | .24 |
| IV | .44 | .34 | .44 | 1.00 | .26 | .39 | .47 | .46 | .29 | .21 | .32 | .28 | .38 | .30 |
| V | .27 | .45 | .33 | .30 | 1.00 | .45 | .39 | .33 | .32 | .36 | .31 | .31 | .16 | .33 |
| VI | .40 | .40 | .44 | .46 | .51 | 1.00 | .45 | .38 | .38 | .33 | .33 | .28 | .34 | .49 |
| VII | .52 | .43 | .46 | .52 | .43 | .50 | 1.00 | .62 | .45 | .54 | .49 | .41 | .39 | .42 |
| VIII | .53 | .51 | .44 | .51 | .38 | .42 | .67 | 1.00 | .54 | .43 | .48 | .43 | .48 | .53 |
| IX | .29 | .48 | .36 | .37 | .36 | .44 | .51 | .62 | 1.00 | .46 | .48 | .36 | .43 | .35 |
| X | .34 | .50 | .46 | .24 | .39 | .39 | .58 | .48 | .52 | 1.00 | .37 | .41 | .25 | .40 |
| XI | .49 | .32 | .41 | .38 | .35 | .38 | .54 | .55 | .55 | .43 | 1.00 | .42 | .39 | .40 |
| XII | .40 | .27 | .15 | .33 | .36 | .34 | .49 | .50 | .41 | .48 | .49 | 1.00 | .31 | .42 |
| XIII | .40 | .28 | .42 | .43 | .18 | .39 | .43 | .55 | .50 | .29 | .45 | .35 | 1.00 | .48 |
| XIV | .44 | .29 | .28 | .33 | .37 | .55 | .47 | .57 | .40 | .44 | .44 | .47 | .53 | 1.00 |

*Note*. ICE-FPSQ = Iceland-Family Perceived Support Questionnaire. Items I – XIV are from the original ICE-FPSQ (Sveinbjarnardottir et al., 2012). Correlations produced using polychoric correlation were compared to those produced when using Pearson correlation.

| The boxes marked with |  | indicate the highest correlation |
| --- | --- | --- |

Table 2

*Model Fit Indices of Pearson CFA and the Polychoric CFA*

| **Parameter** | **Pearson CFA** | **Polychoric CFA** |
| --- | --- | --- |
| RMSEA | .109 (Interval: .102-.116) | .076 (Interval: .069-.084) |
| SRMR | .058* | .059* |
| TLI | .811 | .983* |
| CFI | .842 | .986* |
| Chi-square | *X^2^* = 720.791; df=76, *p*<.001 | *X^2^*= 391.99; df=76; *p*<.001 |
| Estimator | ML | WLSMV |

*Note*. CFI = Comparative Fit Index; RMSEA = Root Mean Square Error Approximation; SRMR = Standardized Root Mean Square Residual; ML: Maximum Likelihood; WLSMV: weight least squares, means, and variances; TLI = Tucker Lewis Index; *X^2^* indicates chi-square.

*Parameters indicate model fit

Table 3

*Family Characteristics Related to Cognitive and Emotional Support*

| **Sample characteristics** | **Estimate** | **Mean Sq** | ***F*-value** | ***p*r(>F)** |
| --- | --- | --- | --- | --- |
| Cognitive support |  | | | |
| Education  None  Primary  Secondary  Post-secondary | 4.50  2.45  1.03  0.89  0.46 | 1.12  0.44  0.28  0.25  0.68 | 2.76  5.55  3.64  3.42  0.67 | .02  <.001  <.001  <.001  .58 |
| Relationship to the patients  Mother  Father  Sibling  Wife  Husband  Friend  Surrogate  Grandparent  Child of patient | 8.12  0.37  2.61  0.32  0.13  0.49  0.53  0.26  0.13  1.04 | 0.90  0.68  0.68  0.19  0.33  0.44  0.24  0.19  0.13  0.40 | 2.21  0.54  3.83  1.64  0.41  1.12  2.21  1.34  0.96  2.60 | .01  .58  <.001  .10  .68  .26  .02  .17  .33  <.01 |
| Education: relationship to the patient **^c^** | 25.77 | 1.43 | 3.51 | <.01 |
| Emotional support |  | | | |
| Education  None  Primary  Secondary  Post-secondary | 3.92  0.47  0.49  0.40  0.07 | 0.98  0.20  0.17  0.16  0.65 | 2.60  2.33  2.90  2.46  0.11 | .03  .02  .003  .014  .91 |
| Relationship to the patient  Mother  Father  Sibling  Wife  Husband  Friend  Surrogate  Grandparent  Child of patient | 7.34  0.10  0.001  0.15  0.13  0.04  0.58  0.13  0.01  0.25 | 0.81  0.13  0.08  0.15  0.29  0.09  0.18  0.17  0.11  0.12 | 2.16  0.78  0.014  1.02  0.45  0.43  3.13  0.76  0.09  2.07 | .02  .43  .98  .30  .65  .66  .001  .44  .92  .03 |
| Education: relationship to patient **^c^** | 24.47 | 1.35 | 3.61 | <.01 |

*Note***.** mean sq = mean square; *pr (>F)* = *p*-value for the F statistic

**^a^** Values reflect the interactions of variables. Further analyses of interaction of variables are provided in SDC Table 3.
