# Supplementary material for: Polychoric Correlation With Ordinal Data in Nursing Research
Source: Nurs Res. 2022 Aug 20;71(6):469–76. doi: 10.1097/NNR.0000000000000614 (PMC9617753; doi:10.1097/NNR.0000000000000614)
Supplement: SUPPLEMENTARY MATERIAL [file nres-71-469-s002.docx]

Supplementary table 1. Factor loading for the ICE-FPSQ (n = 810)

| **ICE-FPSQ items** | **Factors** | |
| --- | --- | --- |
|  | Cognitive support | Emotional support |
| I | 0.69 |  |
| II | 0.66 |  |
| III | 0.66 |  |
| IV | 0.61 |  |
| V | 0.60 |  |
| VI |  | 0.65 |
| VII |  | 0.81 |
| VIII |  | 0.80 |
| IX |  | 0.72 |
| X |  | 0.69 |
| XI |  | 0.71 |
| XII |  | 0.61 |
| XIII |  | 0.65 |
| XIV |  | 0.66 |

Note. ICE-FPSQ = Icelandic-Family Perceived Support Questionnaire. Items I – XIV are from the original ICE-FPSQ (Sveinbjarnardottir et al. 2012).

All loadings are higher than the acceptable threshold level of 0.6 (Field, 2005)

**Supplementary table 2**: Ordinal and Pearson Cronbach alpha values for the ICE-FPSQ (n = 810)

| **ICE-FPSQ items** | **Ordinal Cronbach’s alpha values** | **Pearson Cronbach’s alpha values** | **Difference** |
| --- | --- | --- | --- |
| I | 0.887 | 0.907 | 0.019 |
| II | 0.887 | 0.907 | 0.020 |
| III | 0.889 | 0.908 | 0.019 |
| IV | 0.889 | 0.908 | 0.019 |
| V | 0.891 | 0.910 | 0.019 |
| VI | 0.886 | 0.906 | 0.019 |
| VII | 0.881 | 0.901 | 0.021 |
| VIII | 0.880 | 0.901 | 0.020 |
| IX | 0.885 | 0.905 | 0.020 |
| X | 0.886 | 0.906 | 0.020 |
| XI | 0.885 | 0.905 | 0.020 |
| XII | 0.889 | 0.908 | 0.019 |
| XIII | 0.888 | 0.907 | 0.020 |
| XIV | 0.886 | 0.906 | 0.020 |

Abbreviations: ICE-FPSQ denotes Iceland-family perceived support questionnaire (Sveinbjarnardottir et al. 2012).

Roman numerals I – XIV indicated the fourteen items on the ICE-FPSQ scale.

**Supplementary Table 3:** Interaction effects between background variables with cognitive and emotional support (n = 810)

| Factor information | Estimate | Mean Square | F-value | | pr(>F) | |
| --- | --- | --- | --- | --- | --- | --- |
| **Cognitive support** |  | | | | | |
| Education*****relationship to the patient  No formal education ***** mother of patient  No formal education ***** father of patient  No formal education ***** sibling  No formal education ***** child of patient  Primary education ***** father of patient  Primary education ***** husband of patient  Secondary education ***** sibling | 25.77  2.07  4.21  1.90  2.63  2.8  1.45  2.63 | 1.43  0.80  0.89  0.45  0.70  0.70  0.73  0.68 | 3.51  2.58  4.7  4.18  3.72  3.97  1.98  3.81 | | <0.01  0.009  <0.01  <0.01  <0.01  <0.01  0.04  <0.001 | |
| **Emotional support** |  | | | | | |
| Education ***** relationship to patient | | 24.47 | 1.35 | 3.61 | <0.01 | |
| No formal education * father of patient | | 3.83 | 0.86 | 4.44 | <0.01 | |
| Primary education * father of patient | | 2.76 | 0.67 | 4.08 | <0.01 | |
| Secondary education * father of patient | | 2.44 | 0.66 | 3.69 | 0.0002 | |
| No formal education * sibling of patient | | 1.61 | 0.43 | 3.70 | <0.01 | |
| No formal education *husband of patient | | 1.42 | 0.70 | 2.02 | 0.043 | |
| No formal education * child of patient  No formal education * child of patient | | 2.71  0.87 | 0.67  0.40 | 3.99  2.15 | <0.01  0.03 | |

Note. pr(>F) is the p-value for the F statistic

*Denotes interaction effect of one variable on another variable
